# Alumnæ Association of the Woman’s Medical College of the New York Infirmary

**Published:** 1888-09

**Authors:** 


### ALUMNÆ ASSOCIATION OF THE WOMAN'S MEDICAL COLLEGE OF THE NEW YORK INFIRMARY.

Meeting was held May 29, the President, Dr. Mary T. Bissell, in the Chair.

Dr. Gertrude B. Kelly presented the history of a case of "Acute Articular Rheumatism Complicating the Puerperal State."

The patient was a multipara; the case one of breech presentation. The membranes had been prematurely ruptured at 11 a. m. Dilatation had been completed, and the breech born at 8.45 p. m. Contractions had then ceased, and the head had been delivered by expression. The child was born alive, but did not cry for an hour, and died at the end of twenty-seven hours.

The uterus contracted well, but one hour after labor the pulse was 100, the patient felt chilly, and her face was flushed. On the morning of the second day the temperature was 100.8° Fahr. and the pulse 100. She complained of pain in the right hypochondrium, and epigastric distress. In the evening, the temperature was 102.8° Fahr. and the pulse 104. The face was then of a bluish tint, almost apoplectiform in appearance. The pain and feeling of weight in the epigastrium continued, and a dose of calomel was given. There was no pelvic tenderness, and the flow was normal. On the morning of the third day the temperature was 100.8° Fahr. The tongue was dry and brown, and there was a markedly yellow tinge to the skin, but the patient felt better. On the morning of the fourth day the temperature was 101.4° Fahr., and the pulse eighty-eight. In the evening the temperature was 103° Fahr. and the pulse eighty-eight. Still there was no tenderness about the uterus, and the flow was normal. She now commenced to complain of pain in the pubic symphisis, and in the right elbow. On the morning of the fifth day the temperature was 101.4° Fahr., and there was swelling of the right wrist. The patient was ordered sodæ salicylate, grs. x, q. 2h., but only two doses were taken, because the patient thought that "it stopped the flow." In the evening, the temperature was 99.8° Fahr., perspiration was profuse, and there was not so much pain, but the patient was restless. Following the administration of one drachm of the tincture of hyoscyamus the patient was delirious. She had previously taken this dose with good effect. On the morning of the sixth day the patient was rational and was taking nourishment well. The temperature was 101.4° Fahr. There was now swelling of the left elbow and wrist, but the pain was less. After a fairly comfortable day the patient had, at 3.45, commenced to talk incoherently, and shortly after had died. Embolus was considered to have been the cause of death. No signs of heart implication had been previously discovered. An autopsy was not allowed.

Dr. ELIZABETH CUSHIER favored rather the diagnosis of obscure blood-poisoning in this case. She had seen cases terminate fatally without local pelvic symptoms, and in which on autopsy no pelvic lesions were discovered, with the exception of small collections of pus in the lymph reservoirs situated on the sides of the uterus beneath the broad ligaments. In one case which she had seen, there had been scarcely a trace of peritonitis, and the only changes besides lymphangitis referred to were softening of the liver and spleen, and the dark and semi-coagulable condition of the blood. There was absence of local pelvic symptoms, also, in some cases where the patient after having done apparently well for a few days would have a violent chill and high fever, lasting with more or less marked remissions for a number of days, then passing off and the patient making a rapid and complete recovery. In two such cases seen by the speaker, there was complete absence of local symptoms, and the diagnosis of sepsis could only be made by rigid exclusion. In one such case, the temperature had risen to 107° Fahr. and there was furious delirium. This temperature had been controlled by cold applications and there had been no further elevation.

The joint affection in the case presented by Dr. Kelly, if taken in connection with the delirium, would point to septic rather than rheumatic arthritis, as the cerebral symptoms of acute rheumatism are usually attended or preceded by excessive fever, while in septic poisoning, on the contrary, hyperpyrexia does not necessarily accompany such disturbances.

Dr. EMMA WARD EDWARDS thought that the implication of the joints was too early to be referred to puerperal infection. The joints had been affected upon the fourth day, while the fever had been immediate.

Dr. CUSHIER replied that the poison might have gained access before the child's birth, and in that way have gained time for its work.

Dr. SARAH E. POST suggested that pyosalpinx would furnish such a focus of infective material. Obstetricians were looking toward these tubal collections for the origin of obscure cases of puerperal disease. In this case the head had been delivered by expression. If a tubal collection had existed it might at the same time have been erupted, and passing over the placental surface of the uterus before contraction have been taken directly into the blood. Immediate chill and rise of temperature, under such circumstances, would be thus easily explained. Puerperal infection usually proceeded by the lymphatics. Where the blood current was the channel for its introduction, an altered type of development and speedier systemic involvement would be inferred.

Dr. Virginia Davis, resident physician at the New York Infant Asylum, referred to three cases of joint affection occurring during the puerperal period. In one case the attack had occurred three weeks after delivery, in another two and a half weeks, and in the other, four. The history had been irregular, and the attacks had not been promptly limited by anti-rheumatic treatment, yet the patients had been up and about previous to the attack; there had been no pain nor temperature nor any local pelvic symptoms. A diagnosis of septic infection had been made in all of these cases, but the question of rheumatism was, she thought, pertinent. In one of the cases, a previous history of rheumatism was obtained.

Dr. Kelly closed the discussion. She still held the opinion that the rise of temperature and the joint-affection occurred too early for puerperal pyæmia. In all of the cases which had been referred to, a longer interval had elapsed between the delivery and the development of these symptoms.

Dr. Emma Ward Edwards followed with a paper upon "The Frequency of Backward Displacements of the Uterus after Parturition."

In her earlier practice, she had encouraged patients wearing pessaries for backward displacements to hope that after pregnancy they would be cured. She had, however, been disappointed, having found that in all cases the old malposition was renewed. In this statement she included both flexions and versions. In the reader's experience, all backward displacements of the uterus tended to return after delivery. The curability of the condition was, however, increased. If supported by tampons or a pessary during the first two or three months after delivery, a permanent cure would usually result. The theory of cure lay in a shortening of the ligaments by the processes of involution. Backward displacements in the virgin and in the sterile married woman were, on the other hand, practically incurable by pessaries. The future of Alexander's operation was therefore eagerly watched. It could not be denied that shortening of the round ligaments was the most important indication to be met in the treatment of these cases.

Dr. Grace Peckham referred to a case in which birth at term had been preceded by five miscarriages, due probably to the malposition of the uterus. In this case the malposition had returned after delivery. The special cause for the displacement would, in any case, probably determine its recurrence.

Dr. Post referred to two cases in which posterior displacement had seemed to prevent conception, and pregnancy had occurred while wearing a pessary. In both of these cases the displacement had returned.

Dr. Cushier was sorry to say that she had had a large experience similar to that of which Dr. Edwards had complained. She knew of no case in which backward displacement, existing previous to pregnancy, had not returned. In regard to early treatment, she thought that while careful attention should be paid to the replacing of the uterus as soon after parturition as it was discovered, equal care should be taken that the involution of the vagina was not interfered with by large pessaries. The frequent lifting of the uterus, the Simm's position, or a well-adjusted Cutter's pessary would, she thought, prove most efficient. Later, the intravaginal pessary could be resorted to.

The speaker did not think that backward displacements of the uterus were a frequent cause for sterility, for while she had corrected the displacement in many cases in which it had given rise to repeated abortions, she had yet to see a case of simple retroversion with sterility which was overcome by the replacement of the uterus.

In regard to shortening the round ligaments, her experience had been too limited to be of value. She had operated six times. In three cases the result had been satisfactory; in one, a subsequent operation upon the vagina was resorted to, as the shortening of the ligaments did not prevent the sinking of the uterus; in another the patient was obliged to continue the wearing of her pessary. In one case the operation was done subsequent to a Battey's operation. The round ligaments were found in this case so attenuated as not to bear traction, and relief was given by drawing the cervix backward and attaching its posterior lip to the posterior vaginal wall.

Even where pessaries were worn with comfort, Alexander's operation would be wecolmed as an escape from the doom of wearing them, as was in some cases necessary, for a lifetime.

Dr. SARAH J. MCNUTT made some remarks upon "The Frequency of Nephritis as a Complication of Intestinal Catarrh."

Where stupor or other head-symptoms developed in the course of intestinal catarrh, the urine should be carefully watched. Scanty urine would be often found loaded with albumen and casts.

Dr. GRACE PECKHAM presented a photograph showing the restoration of the parts in a case of the "Tumor of the Clitoris," upon which she had operated one year before.

